# Derivative indices of optic nerve sheath diameter: a novel ultrasound-based approach for non-invasive intracranial pressure monitoring in neurocritical patients

**DOI:** 10.3389/fneur.2026.1942849

**Published:** 2026-09-11

**Authors:** Guangzhao Li, Xiaowang Niu, Kaipeng Zhang, Shanshan Luo, Fei Yang, Xiang Li

**Affiliations:** 1 Department of Neurosurgery, The First People’s Hospital of Hefei, The Third Affiliated Hospital of Anhui Medical University, Hefei, China; 2 Department of Neurosurgery, Suqian Hospital Affiliated to Xuzhou Medical University, Suqian, China; 3 Department of Neurosurgery, Xinghua People’s Hospital Affiliated to Yangzhou University, Xinghua, China

**Keywords:** intracranial pressure, non-invasive intracranial pressure monitoring, optic nerve sheath diameter, preoperative craniotomy assessment, sonography, ultrasound

## Abstract

**Background:**

Preoperative intracranial pressure (ICP) evaluation in neurocritical patients relies on computed tomography (CT) findings, which often lack accuracy. Ultrasound-measured optic nerve sheath diameter (ONSD) offers a non-invasive alternative, but optimal measurement protocols and indices remain controversial.

**Methods:**

We conducted a retrospective cohort study of 45 neurocritical patients undergoing emergency craniotomy with invasive ICP monitoring between January 2023 and December 2025. Preoperative ultrasound measured ONSDint, ONSDext, and 8 derivative indices. Pearson correlation analysis, receiver operating characteristic (ROC) curves, and Kappa consistency tests were used to validate associations with invasive ICP measurements.

**Results:**

All ONSD-derived indices correlated significantly with invasive ICP (*p* < 0.01). Two indices exhibited superior diagnostic performance for identifying ICP ≥ 30 mmHg: ONSDint/OND (AUC = 0.980, sensitivity = 92.6%, specificity = 94.4%, optimal cutoff = 1.56) and ONSDint-OND (AUC = 0.980, sensitivity = 96.3%, specificity = 94.4%, optimal cutoff = 2.0). Kappa analysis showed almost perfect agreement between both indices and invasive monitoring (ONSDint/OND: Kappa = 0.775, accuracy = 88.9%; ONSDint-OND: Kappa = 0.86, accuracy = 93.3%).

**Conclusion:**

ONSDint/OND and ONSDint-OND are reliable non-invasive biomarkers for predicting elevated ICP in neurocritical patients, providing valuable information to guide preoperative surgical decision-making. This study validates the clinical utility of ultrasound-derived ONSD indices in optimizing ICP assessment before craniotomy.

## 1 Introduction

For patients with severe neurological disorders, intracranial pressure (ICP) is routinely assessed before craniotomy based on radiological findings, specifically midline shift and basal cistern compression identified on non-contrast cranial computed tomography (CT) scans. However, this imaging-based assessment lacks sensitivity and specificity, and may not reliably reflect actual ICP values. Compared to CT scanning, Transorbital ultrasound measurement of the optic nerve sheath width (ONSD) is a cost-effective, bedside-performed, dynamic, and radiation-free diagnostic approach for ICP monitoring in critically ill neurologic patients (1–6). Yet, its clinical utility in guiding preoperative decision-making especially regarding optimal timing of surgical intervention remains uncertain. Although an ONSD threshold of >5.0 mm is frequently cited as indicative of elevated ICP (7, 8), this cutoff lacks universal validation. A major barrier to clinical adoption is the absence of standardized acquisition protocols including patient positioning, probe selection, measurement plane and inter-observer training which contributes to substantial interstudy variability and limits generalizability of diagnostic accuracy (9). Among these, determining the optimal measurement distance for ONDS under ultrasound is particularly important.

Currently, two ultrasound-based methods are used to measure the ONSD: the internal diameter (ONSDint)—defined as the distance between the inner margins of the optic nerve sheath (ONS), and the external diameter (ONSDext)—measured between the outer margins. The coexistence of these two approaches largely reflects ongoing debate regarding the sonographic appearance of the subarachnoid space (SAS) surrounding the optic nerve (ON): whether it manifests as hypoechoic or hyperechoic relative to adjacent structures. The SAS filled with cerebrospinal fluid (CSF) is normally hypoechoic on ultrasonography; the presence of hyperechogenicity within this CSF space is suggestive of either a mass lesion or calcification. Recent international consensus guidelines recommend ONSDint as the preferred metric for assessing ONS distension, citing its stronger physiological correlation with ICP elevation (10). However, in routine clinical practice, the ONSDint is often difficult to delineate reliably due to limited ultrasound resolution, acoustic shadowing, or suboptimal probe positioning, rendering its measurement subjective and technically challenging. By contrast, ONSDext tends to be more consistently visualized and reproducibly measured (11). To address this methodological uncertainty and improve preoperative risk stratification, we conducted a retrospective analysis of neurocritical care patients at our institution who underwent invasive ICP monitoring. Specifically, we evaluated the predictive performance of preoperative ultrasound-derived ONSD measurements including both ONSDint and ONSDext, as well as derived indices for detecting elevated ICP (≥30 mmHg). The primary objective was to identify robust, ultrasound-accessible indices that could inform timely surgical decision-making, particularly regarding emergent decompressive interventions.

## 2 Materials and methods

### 2.1 General characteristics of patients with severe neurological conditions

This study conducted a retrospective analysis of 45 patients with severe neurological conditions admitted to The First People’s Hospital of Hefei between January 2023 and December 2025. The inclusion criteria were as follows: (1) CT demonstrating significant intracranial pathology necessitating emergent neurosurgical intervention; (2) Invasive ICP monitoring initiated as the first intraoperative procedure; (3) The ICP catheter was placed in the lateral ventricle; (4) The patient’s guardian provided consent for the ultrasound examination. The exclusion criteria were as follows: (1) Non-ventricular ICP catheter placement (e.g., subdural space, parenchymal parenchyma, or hematoma cavity); (2) Time interval exceeding 60 min between surgical procedure and ONSD measurement via ultrasound; (3) Pre-existing ophthalmic disease, prior trauma to the examined eye, open facial injury, or open head injury with concomitant gross CSF leakage; (4) Pediatric patients aged ≤12 years; (5) Patients with brain tumors complicated by chronic intracranial hypertension. The research protocol was approved by the Ethics Committee of Hefei First People’s Hospital, and informed consent was waived because of its retrospective nature.

### 2.2 ONSD measurement

**Figure 1:**
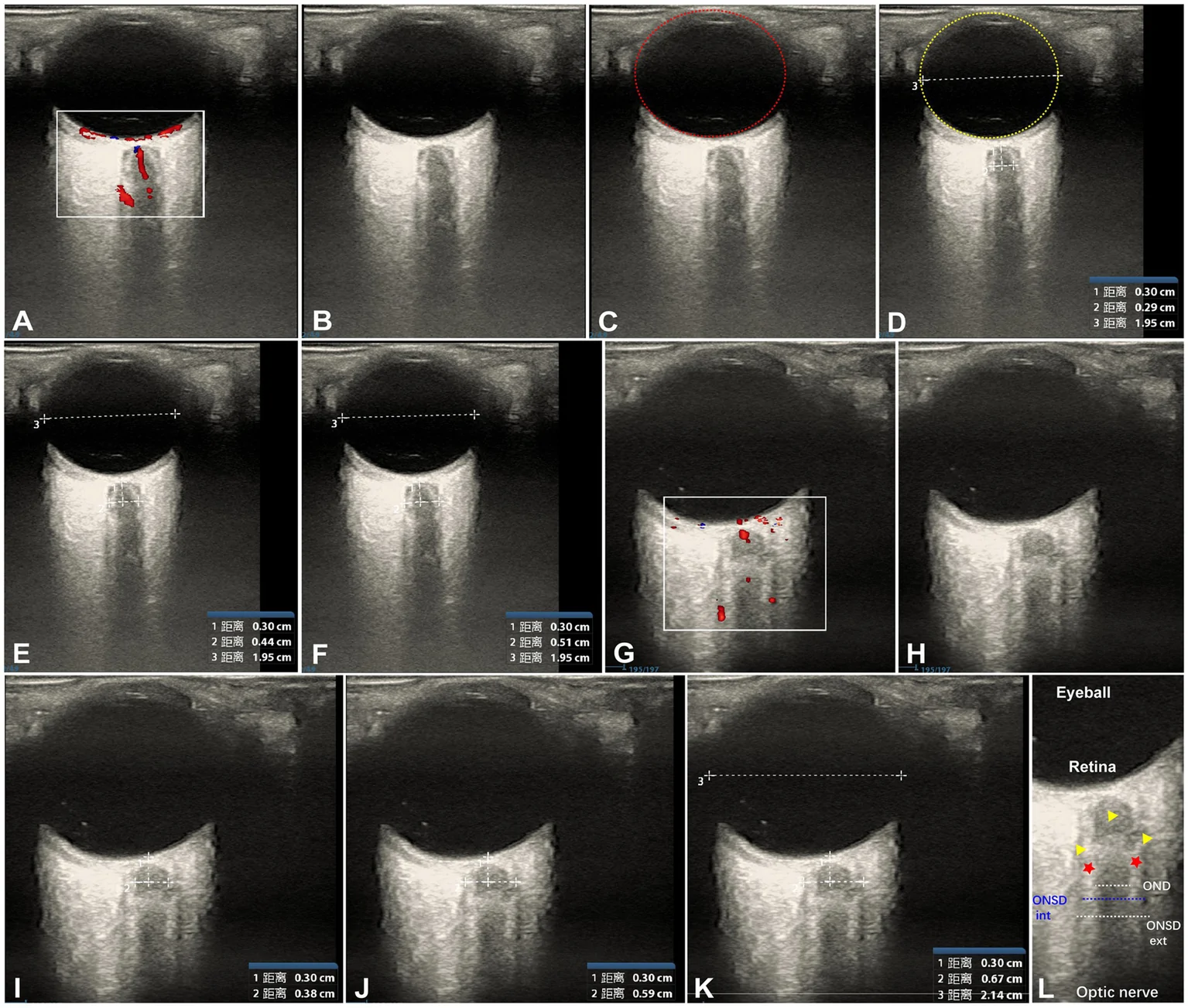
Schematic diagram of ONSD and related parameter measurements. **(A)** Identification of the maximum sheath width under color Doppler mode; **(B)** Elimination of color Doppler imaging; **(C)** Simulation of ocular trajectory; **(D)** Measurement of ONSD; **(E)** Measurement of ONSDint; **(F)** Measurement of ONSDext; **(G–K)** Identical measurement procedure in another patient. **(L)** Optic nerve sheath “three dark, two bright” pattern: schematic illustration where yellow triangles represent three hypoechoic areas and red stars represent two hyperechoic areas. Dashed lines from top to bottom indicate OND, ONSDint, and ONSDext, respectively. ONSD, Optic nerve sheath diameter; ONSDint, The internal ONSD; ONSDext, The external ONSD; OND, Optic nerve diameter.

Immediately after the induction of general anesthesia, ONSD measurements were performed using a Mindray M9 portable ultrasound device, and the procedure was completed before surgical incision disinfection. According to the recommendations of the British Medical Ultrasound Society, for ocular imaging, the thermal index should be ≤1 and the mechanical index should be ≤0.3 (12). The probe contact time to the eye was kept to a minimum, not exceeding 60 s per eye, to avoid any potential thermal or mechanical bioeffects. The procedure was conducted as follows: the patient’s eye was gently cleansed; the eyelid was closed manually; and a sterile ocular protective patch was applied. A generous amount of sterile ultrasound coupling gel was placed over the patch, and a 7.5-MHz linear transducer was positioned gently without applying pressure over the closed eyelid in the axial plane. The operator maintained a neutral wrist position throughout scanning to avoid applying excessive pressure on the eyeball. Color Doppler mode was enabled to identify the optimal axial plane, where the pulsation of the central retinal artery in the central region of the ON could be clearly visualized, and this plane provided the most accurate measurement of the optic nerve diameter (OND). Minor probe adjustments were made to optimize visualization of the sheath boundaries, after which the image was frozen for measurement. Measurements were taken 3 mm posterior to the point where the ON enters the globe, perpendicular to the long axis of the ON. To avoid surgical delay, ONSD assessment was performed exclusively in the eye ipsilateral to the ICP monitoring device. All ultrasounds were performed by neurocritical care physicians with ≥5 years of experience. Three consecutive measurements were obtained per eye, and the mean value was recorded (10) (Figure 1). Measured parameters included: ONSDint and ONSDext; Ratios: ONSDint/OND, ONSDext/OND, ONSDint/eyeball transverse diameter (ETD), and ONSDext/ETD; Absolute differences: (ONSDint-OND) and (ONSDext-OND); Normalized differences: (ONSDint-OND)/OND and (ONSDext-OND)/OND.

### 2.3 ICP monitoring

ICP monitoring was performed using either the Codman® MicroSensor™ ICP Monitor (Johnson & Johnson, USA) or the Spiegelberg ICP Monitor (Spiegelberg GmbH, Germany). Prior to insertion, the transducer at the distal end of the ICP probe was connected to the monitoring display system and zeroed at atmospheric pressure. According to the preoperative surgical plan, the burr hole site was localized 2 cm anterior to the coronal suture and 2.5 cm lateral to the sagittal suture. The ICP probe was advanced into the frontal horn of the lateral ventricle along a trajectory parallel to the sagittal plane, which is defined by an imaginary line connecting the bilateral external auditory meati and directed toward the posterior aspect of the lateral ventricle. Correct intraventricular placement was confirmed by free CSF outflow; once verified, continuous ICP measurement commenced. Subsequently, the stepwise ICP reduction protocol established preoperatively is followed to complete the remainder of the surgery. All patients received postoperative neurocritical care with continuous real-time ICP monitoring; therapeutic interventions were individualized based on dynamic ICP trends and clinical status. The timing of ICP catheter removal was determined clinically, considering sustained ICP normalization (< 20 mmHg for ≥24 h), neurological improvement, and absence of complications. To evaluate the predictive value of ONSD for elevated ICP, patients were dichotomized into two groups according to initial ICP: <30 mmHg versus ≥30 mmHg.

### 2.4 Statistics and analysis

SPSS 23.0 software (IBM Corp., Armonk, NY, USA) was used for data entry, processing, and statistical analysis. Continuous variables are presented as mean±standard deviation (SD). Pearson’s correlation coefficient was calculated to assess the linear association between ultrasonographically measured ONSD, other relevant imaging parameters, and invasively measured ICP. For ultrasound parameters showing statistically significant correlations with ICP, receiver operating characteristic (ROC) curve analysis was performed to determine their diagnostic accuracy including sensitivity, specificity, area under the curve (AUC), and optimal cutoff value using Youden’s index. Inter-rater agreement for ultrasound-based ICP prediction (based on the selected ONSD cutoff) versus gold-standard invasive ICP monitoring was evaluated using Cohen’s kappa statistic. A two-sided *p* < 0.05 was considered statistically significant.

## 3 Results

### 3.1 Baseline characteristics of patients

The baseline characteristics of the 45 enrolled patients are summarized in Table 1. These participants represented the most prevalent clinical subtypes of acute intracranial hypertension. Of the 45 patients, seven initially underwent ventricular drainage on the contralateral side (i.e., opposite to the planned ICP probe insertion site), followed by ICP monitoring catheter placement within 15 min. No patient experienced life-threatening surgical complications.

**Table 1:** Baseline characteristics of patients.

Characteristic	*n* = 45, *n* (%) or mean ± SD
Total number	45 (100.0%)
Sex	
Male	29 (64.4%)
Female	16 (35.6%)
Age (y)	57.24 ± 15.59
Preoperative GCS score	6.3 ± 2.84
Cerebral hernia	
Yes	12 (26.7%)
No	33 (73.3%)
ICP catheter placement site	
Right side	26 (57.8%)
Left side	19 (42.2%)
ICP monitor brand	
Johnson&Johnson	44 (97.8%)
Spielberg	1 (2.2%)
Primary diagnosis	
Traumatic brain injury	15 (33.3%)
Aneurysmal subarachnoid hemorrhage	7 (15.6%)
Intracerebral hemorrhage	6 (13.3%)
Intraventricular hemorrhage	16 (35.6%)
Ischemic stroke	1 (2.2%)

GCS, Glasgow Coma Scale; ICP Intracranial Pressure.

### 3.2 Significant positive correlations between ONSD-derived indices and invasive ICP measurements

**Figure 2:**
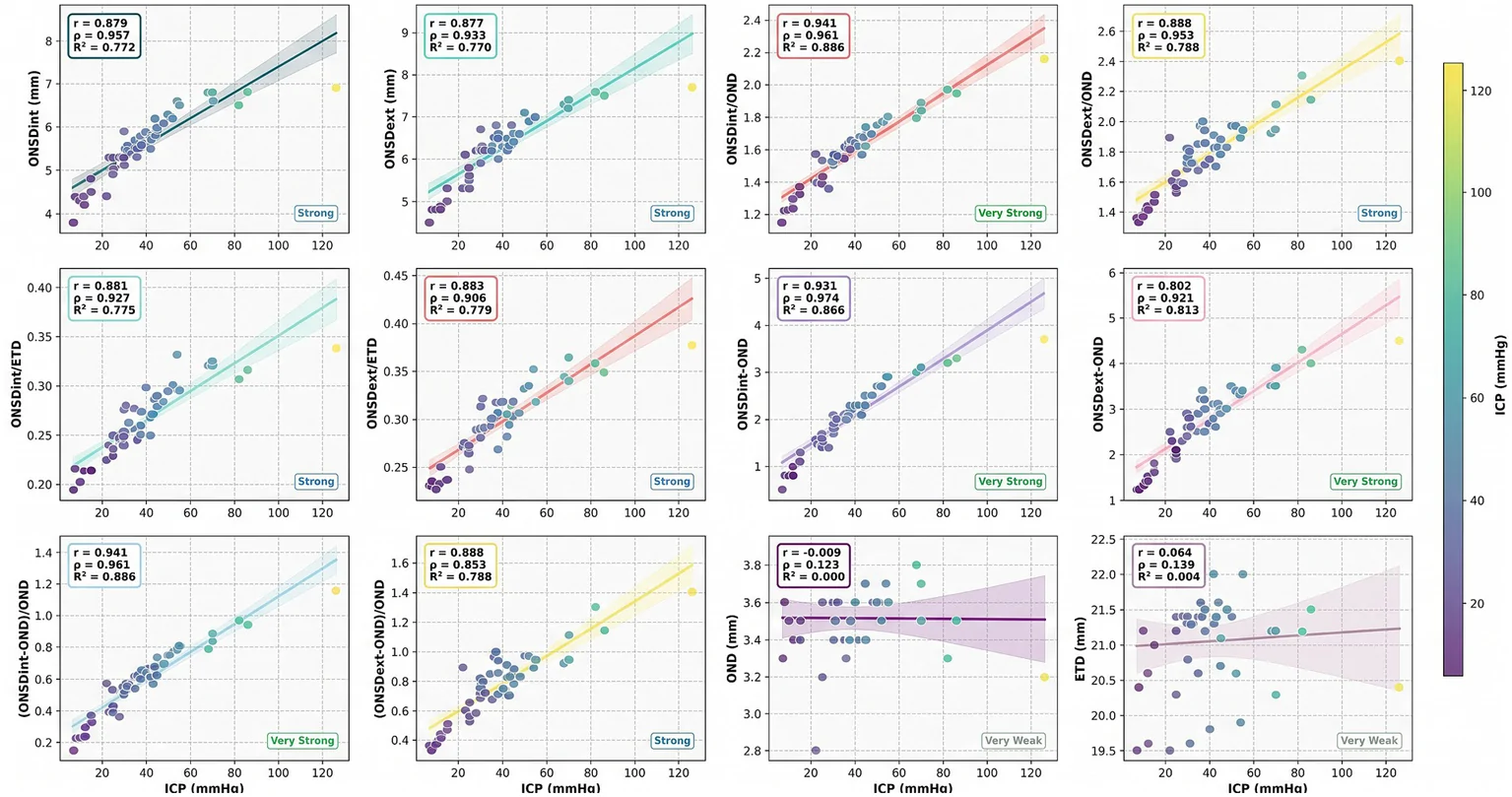
Scatter plot illustrating the correlations between ONSD and its related indicators and ICP. Except for OND and ETD, all other indicators showed significant positive correlations with ICP. Among these, the correlation coefficients were highest for ONSDint/OND and (ONSDint–OND)/OND, followed by ONSDint–OND. ONSD, Optic nerve sheath diameter; ONSDint, The internal ONSD; ONSDext, The external ONSD; OND, Optic nerve diameter; ETD, Eyeball transverse diameter.

With the exception of OND and ETD, both of which did not exhibit a statistically significant correlation with invasively measured ICP, all 10 ONSD-derived indices demonstrated strong positive correlations with ICP (all *p* < 0.001): ONSDint, ONSDext, ONSDint/OND, ONSDext/OND, ONSDint/ETD, ONSDext/ETD, ONSDint-OND, ONSDext-OND, (ONSDint-OND)/OND, and (ONSDext-OND)/OND (Figure 2). Among these, ONSDint/OND and (ONSDint-OND)/OND exhibited the highest correlation coefficients (r = 0.941 and 0.941, respectively), followed closely by ONSDint-OND (r = 0.931). These findings suggest that, within the observed physiological range, increasing values of ONSD and its derived indices are robustly associated with rising ICP.

### 3.3 High diagnostic accuracy of ONSD-derived indices for detecting ICP ≥ 30 mmHg

**Figure 3:**
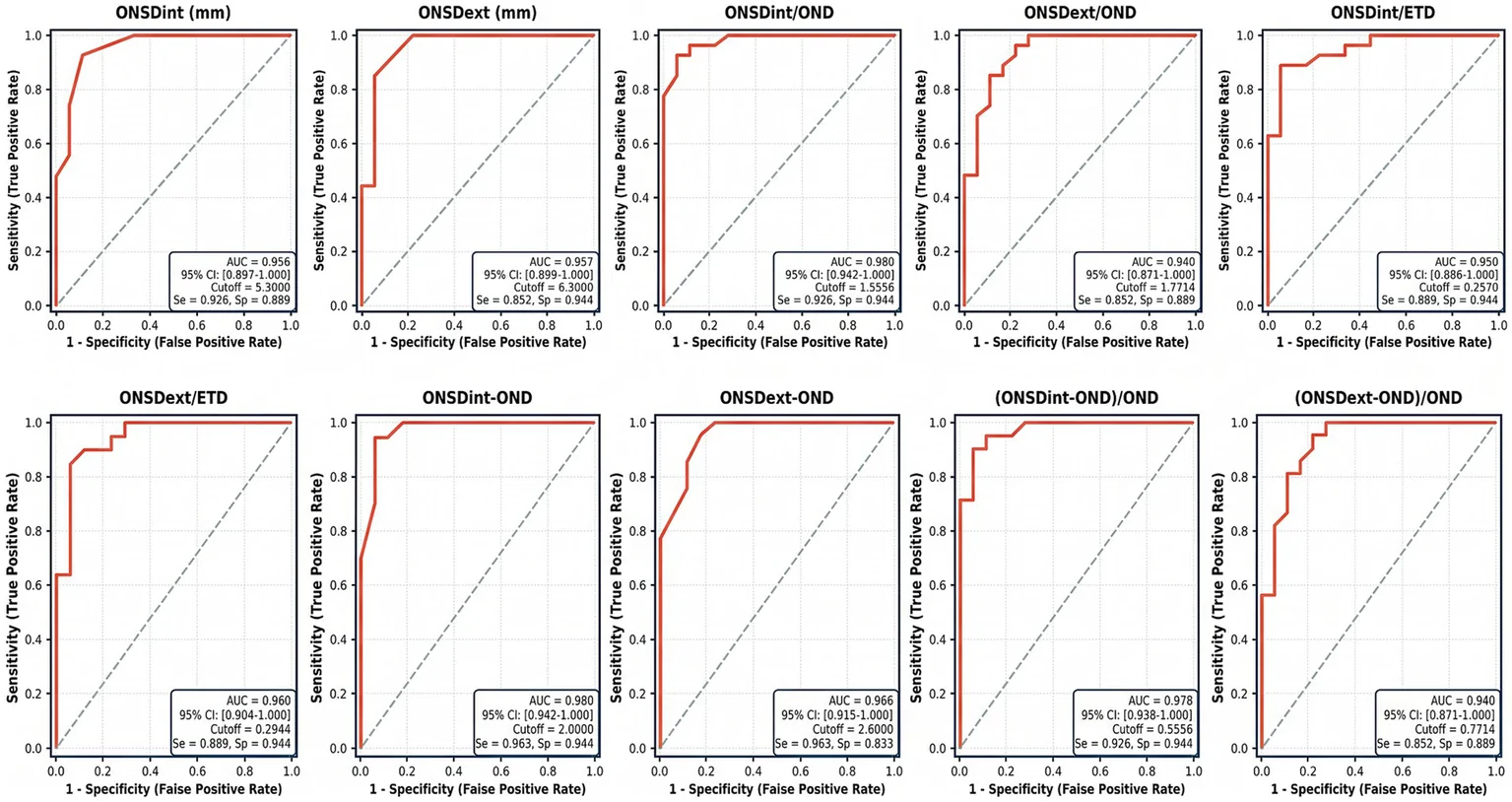
ROC curves of ONSD and its related indicators for diagnosing ICP ≥ 30 mmHg. All 10 associated indicators of ONSD effectively diagnose ICP ≥ 30 mmHg, with ONSDint/OND and ONSDint-OND exhibiting the highest AUC values; all other indicators also demonstrated AUCs above 0.9.\rROC, Receiver operating characteristic; ICP, Intracranial pressure; ONSD, Optic nerve sheath diameter; ONSDint, The internal ONSD; ONSDext, The external ONSD; OND, Optic nerve diameter; ETD, Eyeball transverse diameter; AUC, Area under the curve.

ROC curve analysis was conducted to evaluate the diagnostic performance of the 10 ONSD-derived indices for detecting clinically significant intracranial hypertension (ICP ≥ 30 mmHg). All indices achieved excellent discriminatory capacity, with area under the curve (AUC) values ranging from 0.940 to 0.980. Notably, ONSDint/OND and ONSDint-OND yielded the highest AUCs (0.980 and 0.980, respectively), with corresponding sensitivities of 92.6 and 96.3%, specificities of 94.4% for both, and optimal cutoff values of 1.5556 and 2.00, respectively (Figure 3). Collectively, these results support the use of ONSD-derived indices-particularly ONSDint-OND-as highly reliable, noninvasive surrogates for elevated ICP.

### 3.4 Excellent agreement between ONSDint-OND and invasive ICP monitoring

**Figure 4:**
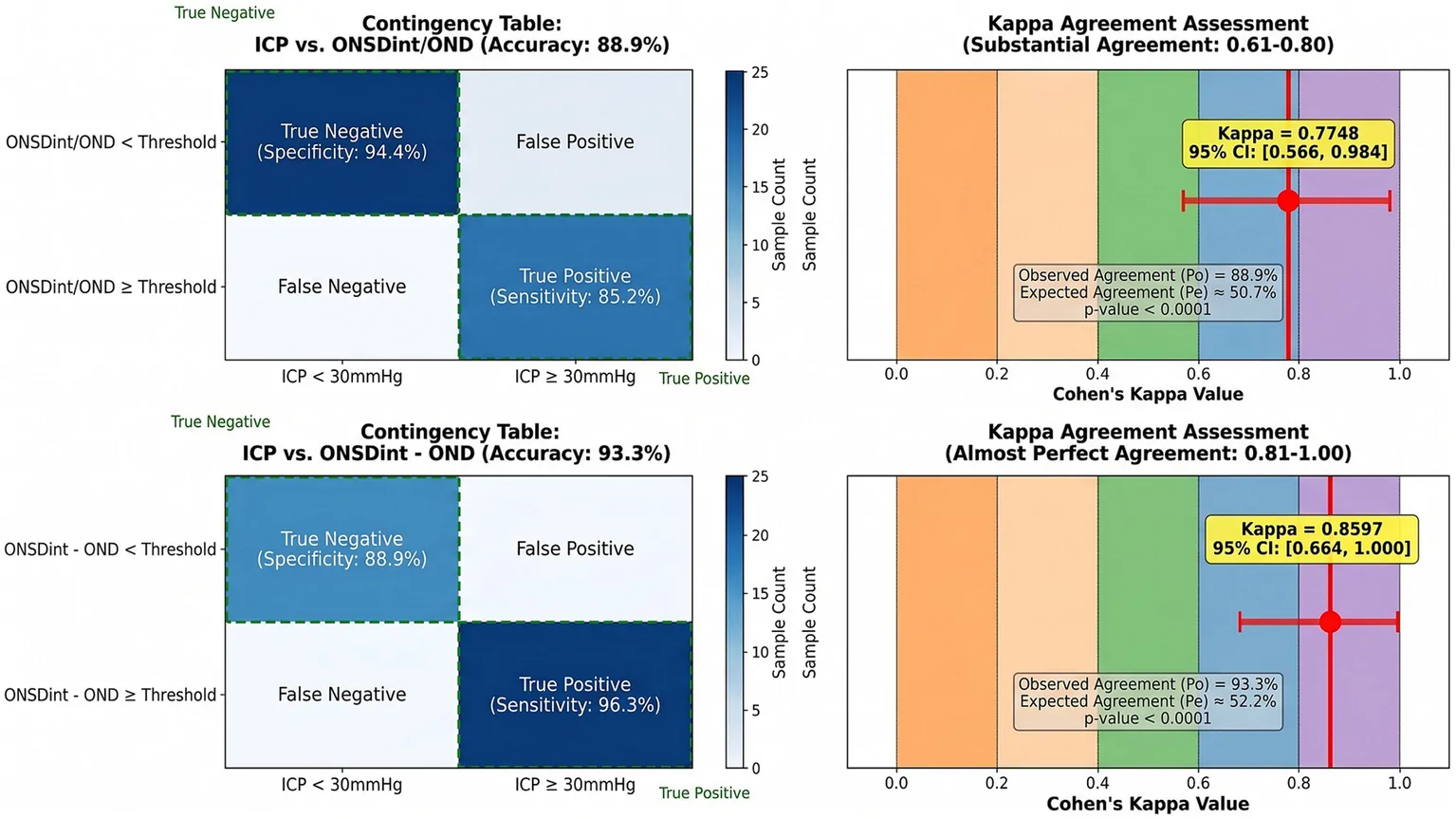
Kappa consistency test between ONSDint/OND and ONSDint-OND in predicting ICP ≥ 30 mmHg compared to the gold standard. The predictive performance of ONSDint/OND and ONSDint-OND for ICP closely matches that of the gold standard, with ONSDint-OND demonstrating superior agreement than ONSDint/OND. ICP, Intracranial pressure; ONSD, Optic nerve sheath diameter; ONSDint, The internal ONSD; ONSDext, The external ONSD; OND, Optic nerve diameter.

To assess clinical concordance, we evaluated the agreement between ONSDint-OND, ONSDint/OND and gold-standard invasive ICP measurements using Cohen’s kappa (κ) and overall accuracy. ONSDint-OND showed near-perfect agreement with invasive ICP monitoring (κ = 0.8597; accuracy = 93.3%), confirming its validity as a noninvasive predictor of elevated ICP. In contrast, ONSDint/OND demonstrated substantial agreement (κ = 0.7621; accuracy = 88.9%) (Figure 4). These findings further reinforce ONSDint-OND as the preferred noninvasive index for rapid, bedside assessment of severe intracranial hypertension.

## 4 Discussion

While obtaining an ultrasound image of the ON is technically straightforward, accurate identification and delineation of the ONS remain challenging (13). On B-mode ultrasound, the ON appears hypoechoic, whereas the surrounding retrobulbar fat is hyperechoic. However, there is currently a lack of consensus on the sonographic interpretation of the intervening hypoechoic and hyperechoic structures between them, particularly with regard to the anatomical correlates of the SAS, pia mater, and dura mater. Some investigators describe the ONS as exhibiting a “three dark, two bright” echogenic pattern: the ON and SAS are hypoechoic; the pia mater (interposed between the ON and SAS) and the dura mater (the outermost meningeal layer, which fuses with retrobulbar fat) are both hyperechoic. Within this framework, ONSD is measured as the distance between the hyperechoic borders of the bilateral retrobulbar fat, which is defined as ONSDext (14, 15). However, Other studies suggest that the SAS is hyperechoic, while the dura mater is hypoechoic. In this interpretation, ONSD should be measured as the distance between the hyperechoic outer margins surrounding the ON, which is also defined as ONSDint. The fundamental difference between the two measurement approaches lies in how the echogenicity of the SAS is interpreted (9, 11, 16). A parallel ambiguity exists in cranial ultrasound: although cerebrospinal fluid (CSF) fills both cerebral sulci/cisterns and ventricles, sulcal and cisternal CSF typically appears hyperechoic, whereas ventricular CSF is anechoic. This difference likely arises from greater acoustic interface density in sulci and cisterns due to arachnoid trabeculae and/or strong reflection at the pia–CSF interface resulting from high acoustic impedance mismatch. Conversely, ventricular CSF exhibits greater volume, homogeneity, and fewer reflective interfaces, yielding weaker echoes. Given the shared anatomical and physiological characteristics between the subarachnoid space surrounding the optic nerve and the subarachnoid space within cerebral sulci and cisterns, it is biologically plausible and has been increasingly supported by evidence that the subarachnoid space within the optic nerve sheath is also hyperechoic (8, 17, 18). This interpretation aligns with current international guidelines, which recommend ONSDint as the primary indicator of ONSD (10). Our study confirms that both ONSDint and ONSDext correlate significantly and strongly with invasively measured ICP. Notably, ONSDint demonstrates superior correlation and is therefore recommended as the primary parameter. Notably, despite ONSDext exhibiting a marginally lower correlation coefficient, it still retains considerable clinical value, especially in cases with suboptimal image quality or indistinct anatomical landmarks, such as emergency and critical care settings. This expands the practical applicability of point-of-care ultrasound for ICP monitoring and supports a more adaptable, context-sensitive measurement strategy. Future research should quantify the diagnostic accuracy, sensitivity, and specificity of each method across diverse pathological states to refine protocol-driven decision-making and enhance early detection of intracranial hypertension, ultimately improving patient safety and outcomes.

ONSD/ETD is widely recognized as a more accurate indicator for predicting ICP (19–21). The key advantage of this indicator is that it accounts for individual anatomical differences in the eye. Ultrasound can clearly visualize the retina, which forms the posterior border of the eyeball. However, due to acoustic shadowing or reverberation artifacts from the lateral and medial walls of the orbit, the medial and lateral borders of the eyeball are sometimes difficult to distinguish from the hyperechoic areas outside the eyeball. Consequently, ETD measurements typically rely on operator-defined virtual boundaries approximated from the globular contour of the globe, introducing substantial inter-observer variability (11). To enhance measurement objectivity and reproducibility, we standardized the axial plane for ONSD assessment as the one in which the central retinal artery is most clearly visualized in color Doppler mode corresponding to the maximal cross-sectional area of the ONS. At this plane, we measured OND instead of ETD as the denominator for normalization. Our analysis revealed that ONSDint/OND, ONSDext/OND, ONSDint-OND, ONSDext-OND, (ONSDint-OND)/OND, and (ONSDext-OND)/OND exhibited statistically significant and strong correlations with ICP elevation (all *p* < 0.001; r ≥ 0.85). Notably, ONSDint/OND and (ONSDint-OND)/OND demonstrated the highest correlation coefficients with ICP (r = 0.941 and r = 0.931, respectively). As corroborated by prior literature, ONSDint/ETD and ONSDext/ETD also showed robust associations with ICP; however, the correlation between ONSDint/OND and ICP was marginally but consistently stronger than that of ONSDint/ETD (r = 0.881), suggesting that OND may serve as a superior individualized denominator. Crucially, OND is anchored to the anatomically stable and sonographically distinct landmark of the central retinal artery, which allows for accurate and reproducible plane localization independent of ambiguous bony contours. Moreover, unlike ETD, OND measurement does not require subjective delineation of virtual ocular boundaries, thereby simplifying acquisition, reducing operator dependency, and enhancing feasibility for routine clinical use. The Fourth Edition of the U. S. Guidelines for the Management of Severe Traumatic Brain Injury (TBI) explicitly states that a stepwise ICP -lowering protocol should be initiated when ICP exceeds 22 mmHg (22). However, this threshold does not automatically indicate the need for immediate surgical intervention. Although decompressive craniotomy for severe TBI is considered to significantly reduce mortality and improve neurological outcomes in such patients, the guideline does not specify at what level of increased ICP decompressive craniotomy should be performed. In the latest Chinese treatment protocol for intracranial pressure management after TBI, surgical intervention (Surgical removal of intracranial hematomas and contusion and laceration or decompressive craniectomy) is recommended when midline shift exceeds 10 mm and ICP is above 30 mmHg (23). Preoperative noncontrast CT scans can indeed detect midline shift and cisternal effacement, which are surrogate radiological markers of elevated ICP. However, definitive quantitative measurement of ICP still relies on invasive monitoring conducted intraoperatively using an intraparenchymal or ventricular ICP probe. Consequently, reliable noninvasive methods to predict ICP preoperatively hold substantial clinical value in guiding timely surgical intervention decisions. To identify optimal predictive indicator, we stratified patients into two groups based on intraoperatively measured ICP < 30 mmHg versus≥30 mmHg. We then evaluated the diagnostic performance of ten candidate ultrasonographic ONSD-related parameters for detecting ICP ≥ 30 mmHg. ROC curve analysis showed that all ONSD-related indicators had significant discriminatory capacity for ICP ≥ 30 mmHg, supporting their utility in preoperative ICP estimation. Among them, ONSDint/OND and ONSDint-OND exhibited the highest AUC values. Further assessment of consistency with the gold-standard invasive ICP measurement demonstrated that both indices achieved excellent concordance. In particular, ONSDint-OND yielded higher weighted kappa and diagnostic accuracy when compared with ONSDint/OND. These findings suggest that ONSDint–OND not only provides robust, operator-independent prediction of clinically relevant ICP elevation but also circumvents potential confounding from nonlinear physiological relationships inherent to ratio-based metrics. Its calculation based on a simple arithmetic difference facilitates intuitive interpretation and straightforward integration into clinical decision thresholds. Accordingly, we recommend ONSDint–OND as the preferred noninvasive sonographic indicator for preoperative risk stratification of ICP ≥ 30 mmHg in neurocritical care settings. It is noteworthy that general anesthesia can indirectly affect intracranial pressure (ICP) by altering cerebral blood flow and cerebral metabolic rate (24). Intravenous anesthetic agents (such as propofol) reduce cerebral metabolism and cerebral blood flow, typically leading to a decrease in ICP, whereas inhalational anesthetics may dilate cerebral blood vessels at high concentrations, potentially resulting in an increase in ICP (25, 26). During general anesthesia, ONSD can also be used for bedside dynamic monitoring of ICP trends; however, its measured values are influenced by factors such as the anesthetic regimen, ventilation status, and intracranial compliance (27). Although general anesthesia may alter both ICP and ONSD, all measurements in this study were conducted under standardized conditions, rendering the impact of general anesthesia on the correlation between ICP and ONSD acceptable.

This study has several limitations. First, the relatively small sample size and single-center design limited the statistical power of subgroup analyses and the generalizability of the results. Second, due to the urgent nature of neurosurgical intervention for patients admitted to the neurocritical care unit, we only measured the eye on the side where the ICP monitor was placed. Although previous studies have reported no significant difference in ONSD between the two eyes, and single-eye measurement is sufficient for routine monitoring purposes (28). Bilateral assessment remains preferable to enhance reliability and mitigate potential laterality-related bias. Third, measurement variability is inevitable and may stem from operator experience, ocular positioning during scanning, and heterogeneity in underlying neurological conditions. Fourth, although all ultrasound measurements were performed by a fixed operator with more than 5 years of experience following a standardized protocol, no formal inter-observer or intra-observer reliability assessment was conducted. Consequently, the reproducibility of the ONSD measurements could not be quantitatively verified, which may somewhat affect the interpretation of the results. Future research will focus on the following improvements: conducting multicenter studies with larger sample sizes, expanding the postoperative time points for ultrasound monitoring of ONSD, and adding simultaneous measurement of the middle cerebral artery pulsatility index. In addition, operators with different levels of experience will be included to perform repeated measurements, thereby strengthening clinical generalizability and methodological rigor.

## 5 Conclusion

Our preliminary study demonstrates that ultrasound measurement indices centered on ONSDint and ONSDext can accurately predict elevated ICP. Among the evaluated indice, ONSDint-OND and ONSDint/OND indices exhibit superior diagnostic performance and hold promise as reliable, noninvasive surrogates for ICP monitoring. They can be applied preoperatively to predict ICP levels in patients undergoing neurocritical care, thus providing a reliable basis for surgical decision-making.

## Data Availability

The original contributions presented in the study are included in the article/supplementary material, further inquiries can be directed to the corresponding author.
